# Association of PNPLA3 rs738409 G/C gene polymorphism with nonalcoholic fatty liver disease in children: a meta-analysis

**DOI:** 10.1186/s12881-020-01098-8

**Published:** 2020-08-18

**Authors:** Shan Tang, Jing Zhang, Ting Ting Mei, Hai Qing Guo, Xin Huan Wei, Wen Yan Zhang, Ya Li Liu, Shan Liang, Zuo Peng Fan, Li Xia Ma, Wei Lin, Yi Rong Liu, Li Xia Qiu, Hai Bin Yu

**Affiliations:** Department of Hepatitis C and Drug-Induced Liver Injury, Beijing YouAn Hospital, Capital Medical University, Beijing, 100069 China

**Keywords:** PNPLA3, Meta-analysis, Polymorphism

## Abstract

**Background:**

Nonalcoholic fatty liver disease (NAFLD) is one of the most common causes of chronic liver disease worldwide. Current studies have shown that PNPLA3 (Patatin-like phospholipase domain containing 3) rs738409 G/C gene polymorphism is associated with adult nonalcoholic fatty liver disease [1, 2].But there is no consensus on the relationship between PNPLA3 rs738409 G/C gene polymorphism and children NAFLD due to differences in population samples. To this end, a meta-analysis of published research is conducted to comprehensively assess the relationship between PNPLA3 gene polymorphism and NAFLD in children.

**Methods:**

We searched MEDLINE, PubMed, EMBASE, and CENTRAL databases from inception to May 2019. Case-control studies assessing the relationship between PNPLA3 rs738409 G/C gene polymorphism with non-alcoholic fatty liver disease in children were selected according to inclusion and exclusion criteria. Random effects model was used to quantify the association between the PNPLA3 rs738409 G/C gene polymorphism and the susceptibility of children’s NAFLD. Fixed effects model was used to quantify the relationship between the PNPLA3 rs738409 G/C gene polymorphism and the severity of NAFLD in children. The Stata 12.0 software was employed for data analysis.

**Results:**

A total of nine case-control studies were included in this meta-analysis containing data of 1173 children with NAFLD and 1792 healthy controls. Five studies compared NAFLD children and non-NAFLD healthy populations. Statistical analysis showed that PNPLA3 gene polymorphism was significantly associated with children’s NAFLD in the allele contrast, dominant, recessive and over dominant models (G vs C,OR = 3.343, 95% CI = 1.524–7.334; GG + GC vs CC,OR = 3.157, 95% CI = 1.446–6.892;GG vs GC + CC,OR = 5.692, 95% CI = 1.941–16.689; GG + CC vs GC,OR = 2.756, 95% CI = 1.729–4.392). Four case-control studies compared Children with nonalcoholic fatty liver (NAFL) and children with nonalcoholic steatohepatitis (NASH). The results showed that the PNPLA3 gene polymorphism was also significantly associated with the severity of NAFLD in children in recessive gene model (GG vs GC + CC,OR = 14.43, 95% CI = 5.985–34.997); The Egger’s test revealed no significant publication bias.

**Conclusions:**

Meta-analysis showed that PNPLA3 gene polymorphism was significantly associated with susceptibility and severity of NAFLD in children.

## Background

Nonalcoholic fatty liver disease (NAFLD) is one of the most common causes of chronic liver disease worldwide. The prevalence of NAFLD in children has reached about 10%, and in obese children has reached 40–70% [3].NAFLD includes nonalcoholic fatty liver (NAFL), nonalcoholic steatohepatitis (NASH),fatty liver fibrosis and cirrhosis. NAFL usually has no obvious clinical symptoms, but hepatocyte injury can lead to the occurrence of NASH, liver fibrosis and cirrhosis in 3–5% of patients [4]. Therefore, assessing the genetic factors of children’s NAFLD to early diagnose the disease, early judge the severity of the disease is the key to improve prognosis. Current studies have shown that PNPLA3 (Patatin-like phospholipase domain containing 3) rs738409 G/C gene polymorphism is associated with adult nonalcoholic fatty liver disease [1, 2].But there is no consensus on the relationship between PNPLA3 rs738409 G/C gene polymorphism and children NAFLD due to differences in population samples, detection methods and diagnostic criteria. To this end, a meta-analysis of published research is conducted to comprehensively assess the relationship between PNPLA3 gene polymorphism and NAFLD in children.

## Methods

The authors performed the meta-analysis according to the Preferred Reporting Items for Systematic Reviews and Meta-Analyses (PRISMA). The meta analysis was registered in PROSPERO. (PROSPERO CRD42019134056).

### Study strategy

MEDLINE、PubMed、EMBASE and CENTRAL were used for the potential studies searching. (NAFLD OR Non alcoholic Fatty Liver Disease OR Nonalcoholic Fatty Liver Disease OR Nonalcoholic Fatty Liver OR Nonalcoholic Fatty Livers OR Nonalcoholic Steatohepatitis OR Nonalcoholic Steatohepatitides) AND (PNPLA3 OR patatin-like phospholipase domain-containing 3) AND (“Child”OR “Adolescent” OR child OR children OR Adolescent* OR student*)were used as the key words or Mesh terms for searching. The final search was conducted up to May 2019.

### Selection of studies

Any study included in current meta-analysis must satisfy the following criteria:1) the study cohorts include PNPLA3 rs738409 G/C gene polymorphism in children with NAFLD and non-NAFLD individuals;2) case-control studies;3) if duplicate research reports was retrieved, the most comprehensive one was selected to avoid repeated statistics. 4) the study contained sufficient data of genotype frequency.Articles were excluded based on the following criteria: 1) studies without controls groups;2) the enrolled case groups are not children patients;3) studies without specific distribution of genotype;4) studies reported animal models.

### Data extraction and analysis

Two reviewers independently assessed and reviewed all identified studies in terms of inclusion and exclusion criteria. Conflicts were reached to agreement via the discussion with the third authors.And they extracted data from the original publications: author, year of publication, country of origin, design of the trial, total numbers of patients in the case and control groups, sex ratio, body mass index (BMI), the numbers of cases and controls with the C/C, C/G, and G/G genotypes, whether genotype distribution was consistent with the Hardy-Weinberg equilibrium (HWE). This meta-analysis preformed the data analyzing by using Stata version 12.0.The association of the G/C polymorphism in the PNPLA3 gene with NAFLD in children susceptibility was evaluated in the allelic, dominant, recessive, and super-dominant models. The association of the G/C polymorphism in the PNPLA3 gene with NASH in children susceptibility was evaluated in the recessive model. The results were evaluated using the OR value and the corresponding 95% confidence interval (CI). The evaluation of heterogeneity of studies was using the *Q* test and was quantified using *I*^2^. The fixed-effects model was used when *I*^2^  <  50%. In contrast, the random-effects model was applied when *I*^2^  >  50%.The suitability test was used to check whether the gene distribution of the control group consistent with the Hardy-Weinberg equilibrium, and the P_H-W_ > 0.05 was regarded as consistent with the HWE.The sensitivity analysis was performed by assessing the change of combined ORs values after elimination of each individual study.The publication bias was evaluated using the Egger linear regression method and funnel chart.

## Results

### Characteristics of the included studies

**Fig. 1:**
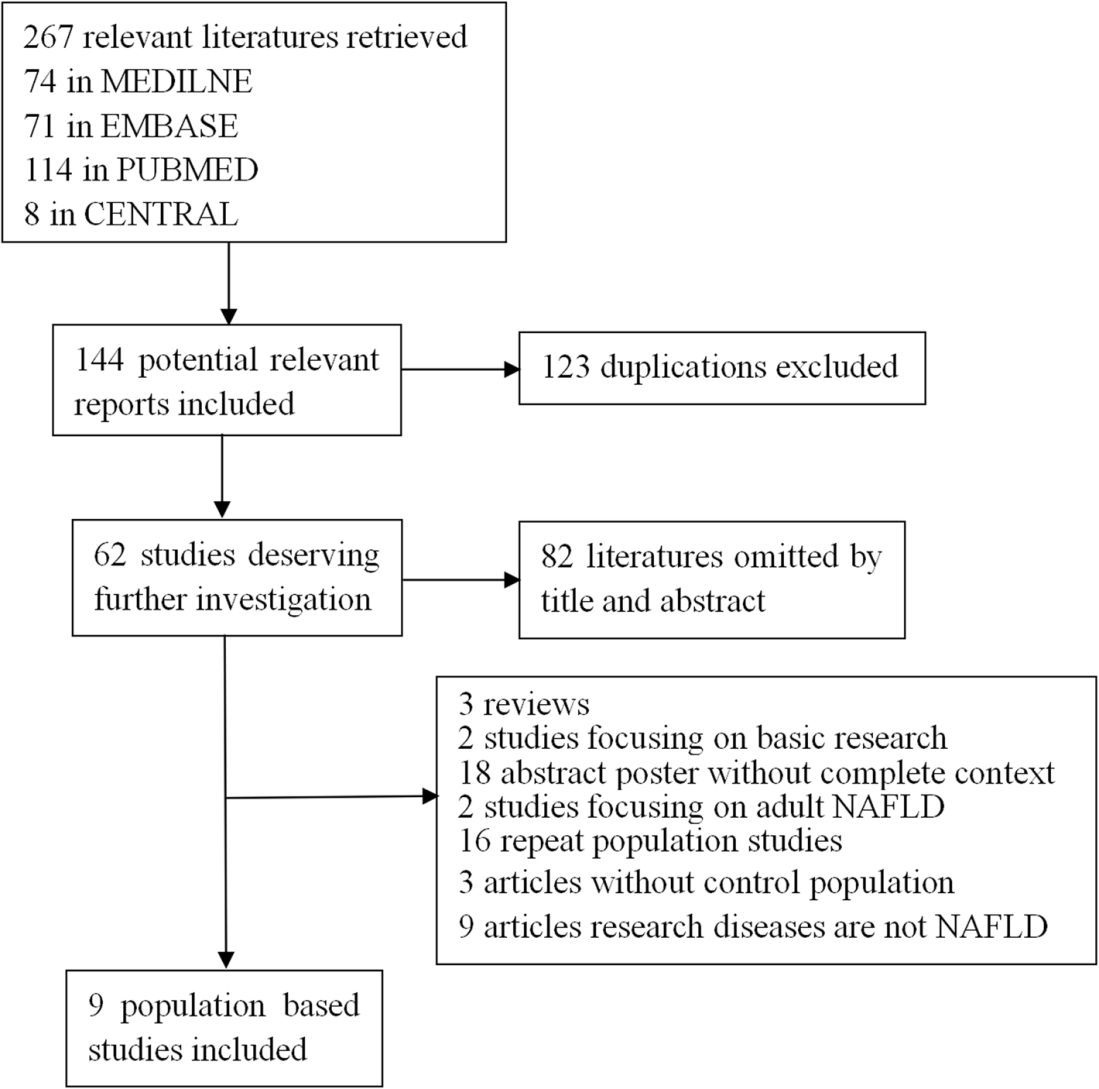
Flow diagram for study selection

The flow diagram of study selection was presented in Fig. 1.According to the set search formula, 267 articles that meet the requirements were initially retrieved, and 123 duplicate articles were removed. 82 unrelated documents were excluded after reading the title and abstract.Then 53 articles were excluded after reading the full text leaving 9 articles that were included in the final analysis. 5 literatures described the association between PNPLA3 rs738409 G/C gene polymorphism and susceptibility to childhood NAFLD disease. There were 550 children with NAFLD and 1792 non-NAFLD controls.4 articles described the association between PNPLA3 rs738409 G/C gene polymorphism and the severity of NAFLD in children. There were 383 children with NASH as case group and 240 children with NAFL control as control group contained in this meta analysis.Genotyping data and basic characteristics for all studies are summarized in Table 1.

**Table 1:** Characteristics of the studies included in the meta-analysis

First author, year, study	Cohort characteristics	Country	Gender (men/women)	BMI (mean ± SD)	Study design	Genotyping	Sample size	genotype			allele		HWE	P_H-W_
								CC	GC	GG	G	C		
Sood.V 2018 [5]	NAFLD children	India			case control study		69	22	23	24	71	67	no	0.005
	NON-NAFLD						30	21	8	1	10	50	yes	0.82
Stasinou.E 2018 [6]	NAFLD children	Greece			case control study	RFLP	58	8	10	40	90	26	no	0.0001
	NON-NAFLD						91	66	22	3	28	154	yes	0.49
Hudert.C 2018 [7]	NAFLD children	German	54/16	35.9 ± 14.5	case control study	TaqMan	70	20	31	19	69	71	yes	0.339
	NON-NAFLD		123/77				200	118	71	11	93	307	yes	0.94
Lin.Y-C 2014 [8]	NAFLD children	China	555/242	27.0 ± 4.1	case control study	TaqMan	191	58	95	38	171	211	yes	0.93
	NON-NAFLD		115/47	26.75 ± 3.85			606	238	293	75	443	769	yes	0.29
Shang.X-R 2015 [9]	NAFLD children	China	459/406	20.72 ± 3.61	case control study	Mass ARRAY	162	60	74	28	130	194	yes	0.53
	NON-NAFLD			28.8 ± 2.8			865	338	418	109	636	1094	yes	0.24
Bugianesi.E 2017 [10]	NASH	Italy		28.2 ± 3.5	case control study	TaqMan	219	74	101	44	189	249	yes	0.37
	NAFL		41/30	95 ± 6			69	36	30	3	36	102	yes	0.29
Nobili.V 2014 [11]	NASH	Italy	51/30	95 ± 6	case control study	TaqMan	71	48	23					
	NAFL		93/56				81	80	1					
Valenti.L 2010 [12]	NASH	Italy			case control study	TaqMan	69	1	44	23	90	46	no	0.0002
	NAFL			28.2 ± 3.6			81	64	17	0	17	145	yes	0.29
Mosca.A 2018 [13]	NASH	Italy		27.8 ± 6	case control study	TaqMan	25	5	13	7	27	23	yes	0.82
	NAFL						9	9	0	0	0	18		

### Meta-analysis results

**Fig. 2:**
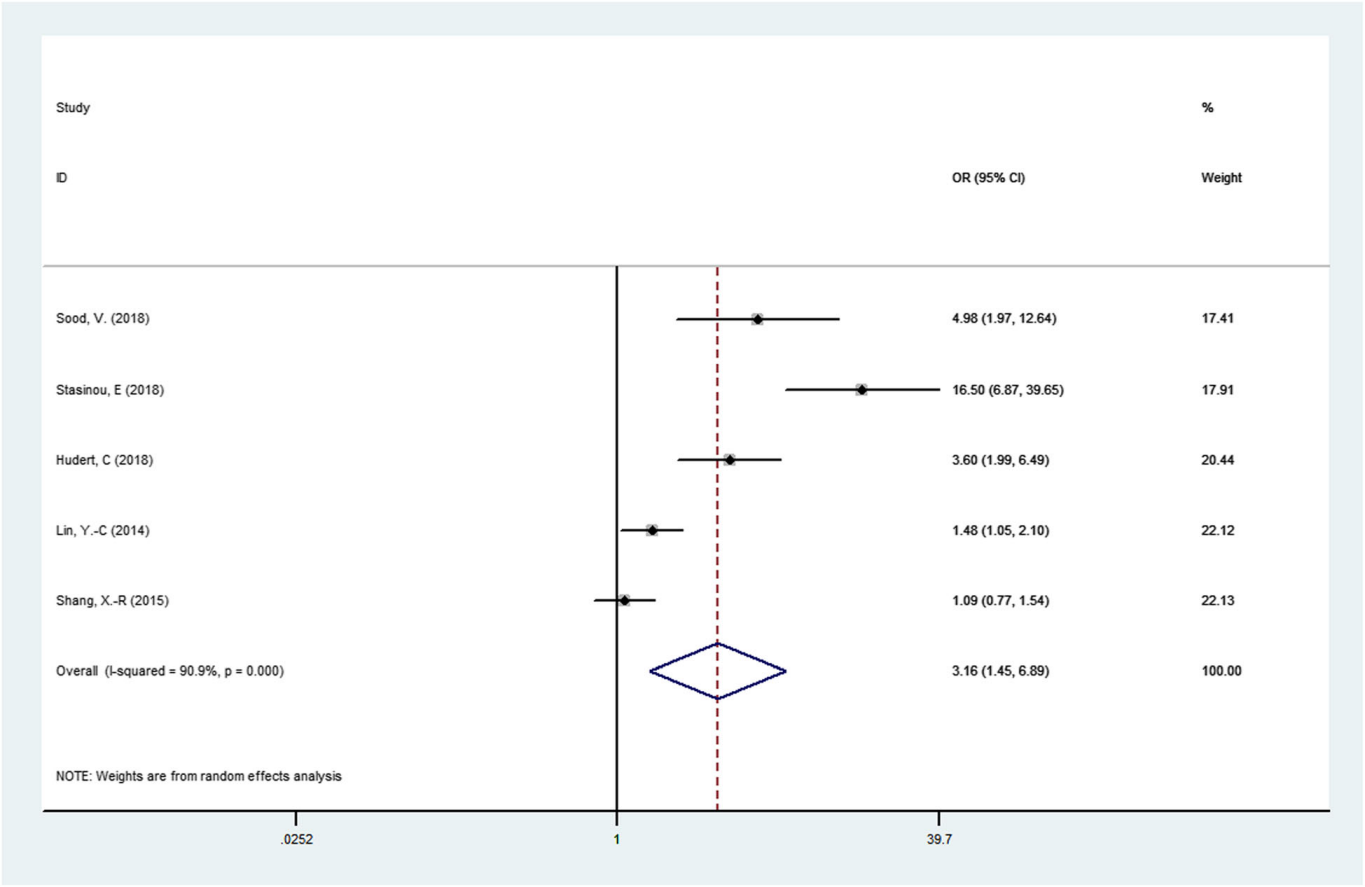
Forest plot of studies evaluating the OR with 95%CI of PNPLA3 rs738409 G/C in the dominant model(GG + GC vs CC) in NAFLD children. CI, Confidence interval; OR, odds ratio

**Fig. 3:**
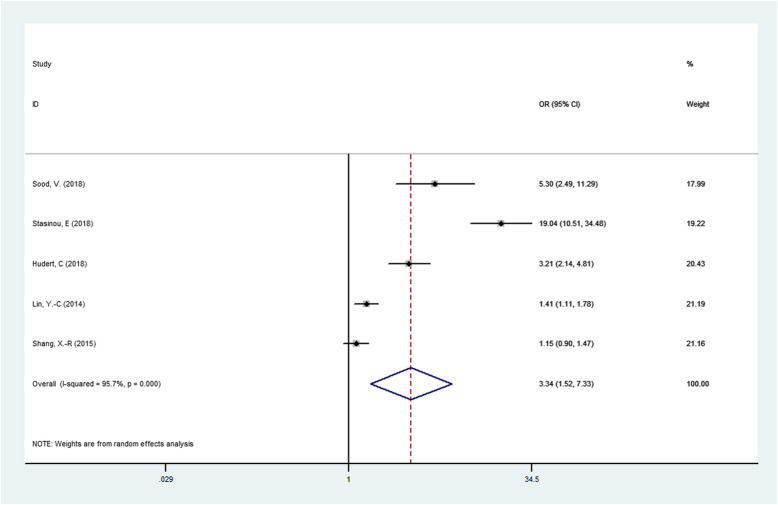
Forest plot of studies evaluating the OR with 95%CI of PNPLA3 rs738409 G/C in the allele model (G vs C) in NAFLD children. CI, Confidence interval; OR, odds ratio

**Fig. 4:**
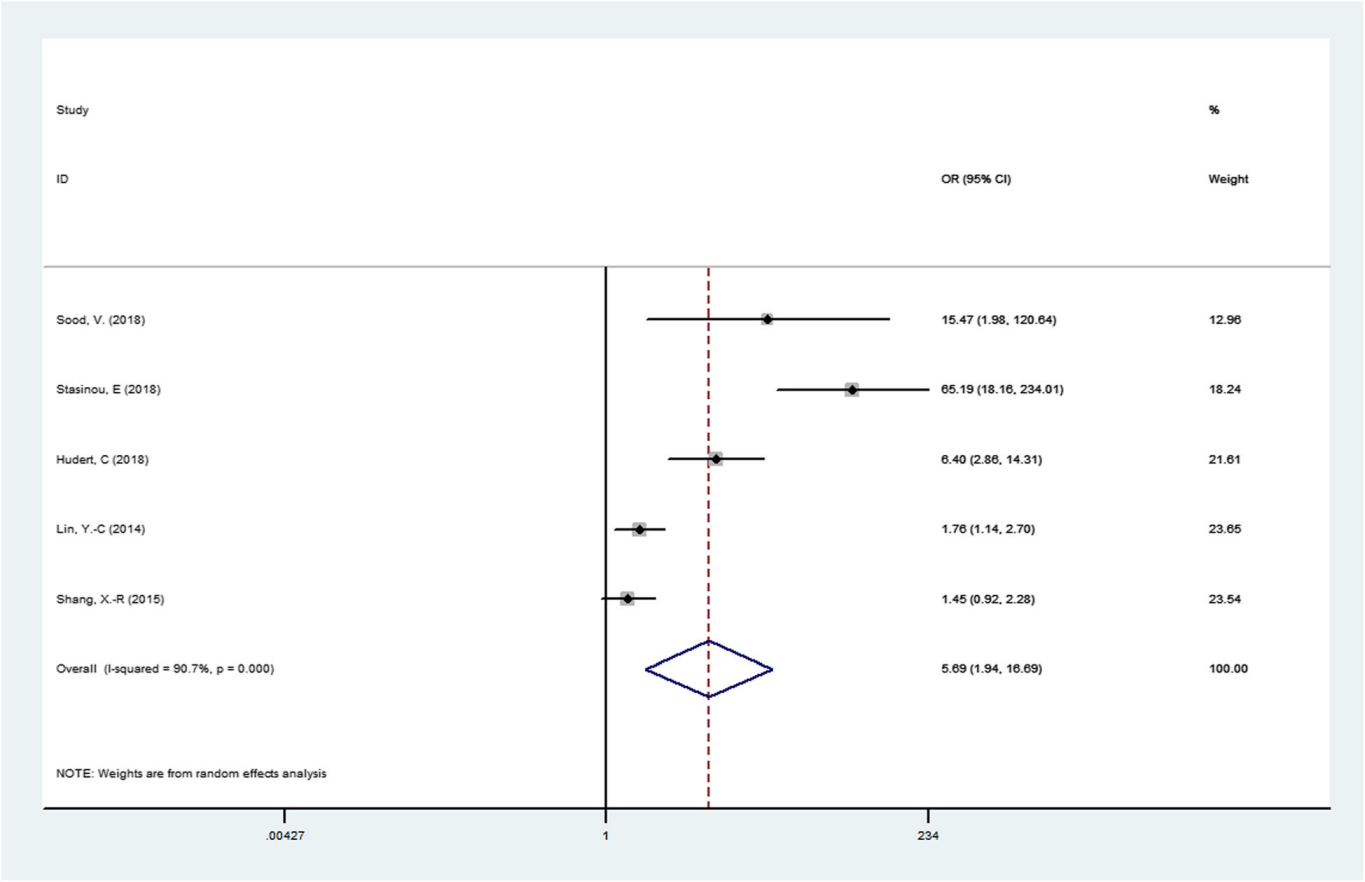
Forest plot of studies evaluating the OR with 95%CI of PNPLA3 rs738409 G/C in the recessive gene model (GG vs CG + CC) in NAFLD children. CI, Confidence interval; OR, odds ratio

**Fig. 5:**
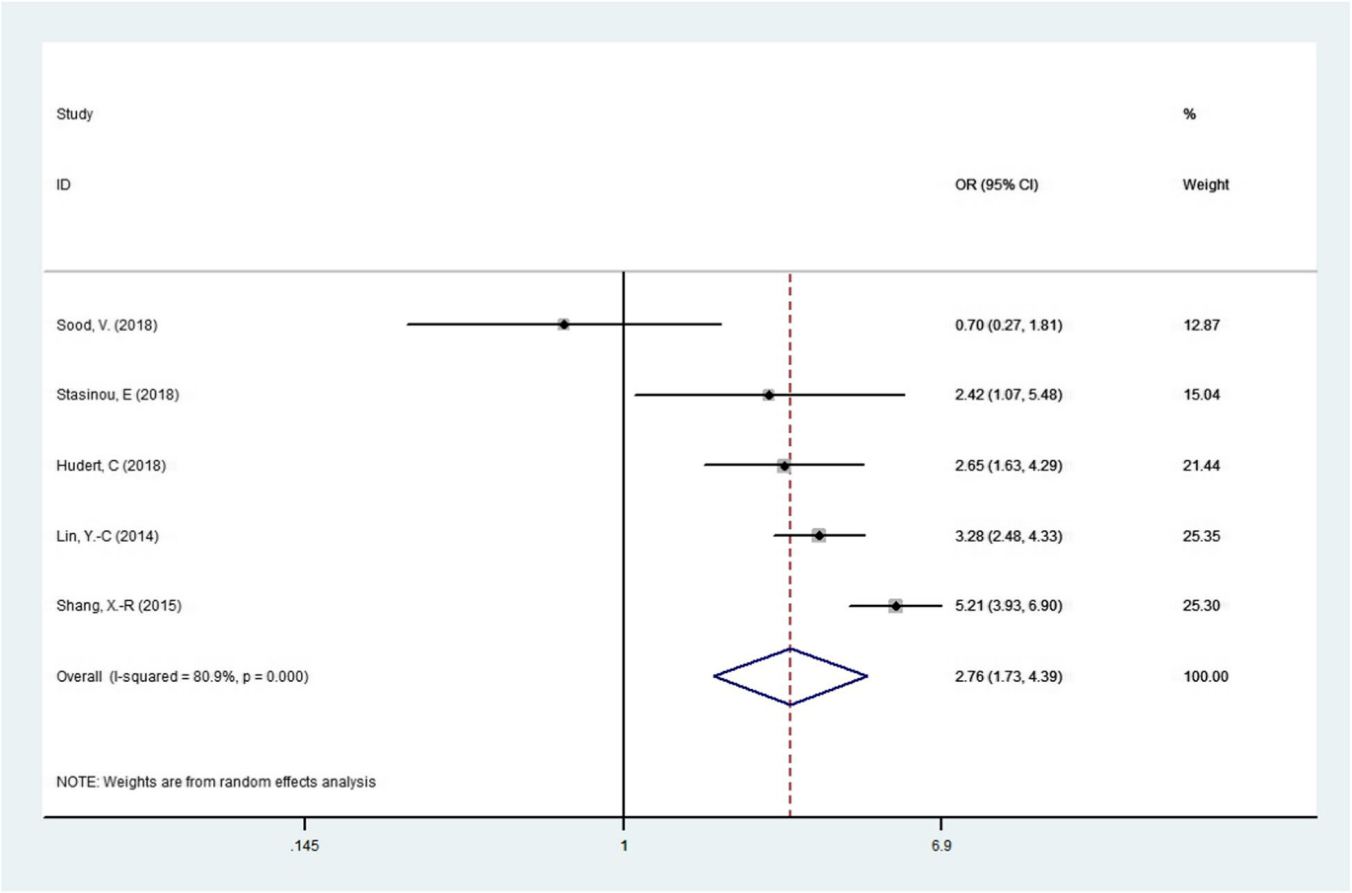
Forest plot of studies evaluating the OR with 95%CI of PNPLA3 rs738409 G/C in the superdominant model (GG + CC vs GC) in NAFLD children. CI, Confidence interval; OR, odds ratio

The results of association between PNPLA3 rs738409 G/C gene polymorphism and susceptibility to NAFLD in children were presented in Table 2. We found that the PNPLA3 rs738409 G/C gene polymorphism was associated NAFLD in children under four genetic models.(For GG + GC vs CC OR = 3.157, 95% CI = 1.446–6.892, *P* = 0.004; Fig. 2.For G vs C OR = 3.343, 95% CI = 1.524–7.334, *P* = 0.003;Fig. 3.For GG vs GC + CC OR = 5.692, 95% CI = 1.941–16.689, *P* = 0.002;Fig. 4.For GG + CC vs GC OR = 2.756, 95% CI = 1.729–4.392, *P* = 0.000;Fig. 5, respectively),suggesting G allele was a risk factor for NAFLD risk in children.

**Table 2:** Meta-analysis of the association of PNPLA3 rs738409 G/C gene polymorphism and childhood NAFLD susceptibility

Genetic model	Relevance test			Heterogeneity test			Publication	
	OR(95% CI)	Z	*P*_value_	I^2^	Q	P_hel_	P_egger_	t
dominant gene model	3.157 (1.446–6.892)	2.89	0.004	90.9	43.75	0.000	0.060	2.95
Allelic model	3.343 (1.524–7.334)	3.01	0.003	95.7	93.77	0.000	0.081	2.59
recessive model	5.692 (1.941–16.689)	3.17	0.002	90.7	42.91	0.000	0.076	2.67
super-dominant model	2.756 (1.729–4.392)	4.26	0.000	80.9	20.93	0.000	0.038	−3.56

**Fig. 6:**
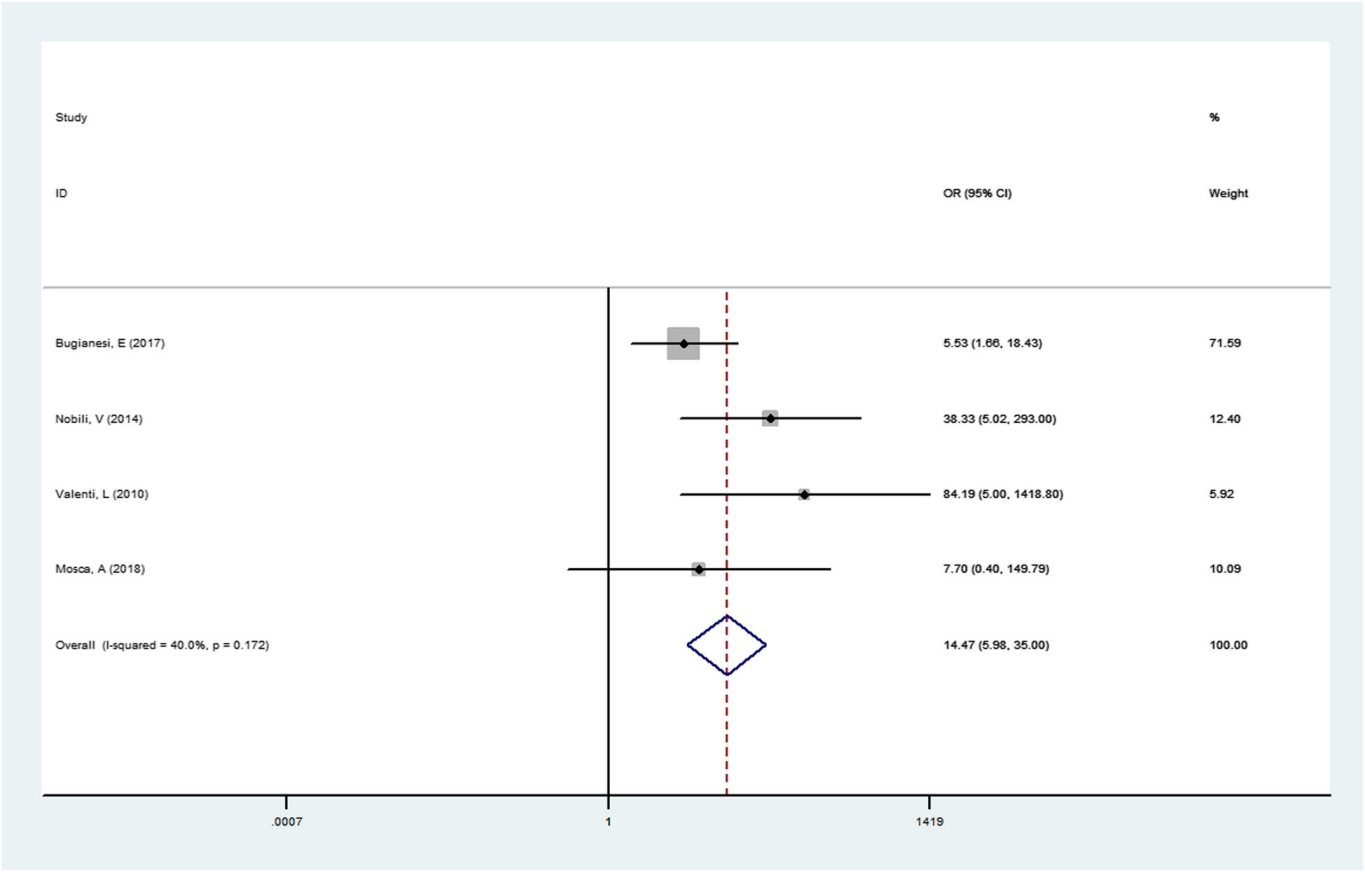
Forest plot of studies evaluating the OR with 95%CI of PNPLA3 rs738409 G/C in the recessive gene model (GG vs CG + CC) in NASH children. CI, Confidence interval; OR, odds ratio

The result of association between PNPLA3 rs738409 G/C gene polymorphism and susceptibility to NAFLD in children were presented in Table 3.The result showed that the risk of NASH in NAFLD patients with GG genotype was higher than that in GC + CC genotypes. (GG vs GC + CC OR = 14.473, 95% CI = 5.985–34.997, *P* = 0.000; Fig. 6).

**Table 3:** Meta-analysis of the association of PNPLA3 rs738409 G/C gene polymorphism and childhood NASH susceptibility

Genetic model	Relevance test			Heterogeneity test			Publication	
	OR(95% CI)	Z	*P*_value_	I^2^	Q	P_hel_	P_egger_	t
recessive model	14.473 (5.985–34.997)	5.93	0.000	40.0	5.00	0.172	0.602	0.61

### Sensitivity analysis and publication bias

To confirm the stability of above results, sensitivity analysis was performed by omitting one study sequentially to examine its effect on the overall results under all genetic models.The result did not alter after deleting each study, indicating the stability of the results of this meta-analysis. (Supplementary Figures 1, 2, 3, 4, 5).Publication bias of the recruited studies was evaluated using Egger regression analysis and funnel chart, the Egger regression analysis and funnel chart suggested that no evidence of publication bias in this meta-analysis.(Supplementary Figs. 6, 7, 8,9,10,11,12,13,14,15).

## Discussion

The phospholipase domain protein 3(PNPLA3) was originally cloned from the cDNA library of 3 T3 preadipocytes into mature adipocytes, hence named adiponectin [14]. It is 10 times more expressed in the liver than in adipose tissue [15]. Non-synonymous genetic variation (rs738409) in the human patatin-like phospholipase domain-containing 3 gene (PNPLA3) that substitutes methionine for isoleucine (I148M) at amino acid position 148 in exon 3 was found to be associated with remodeling of liver triglycerides [16]. Hepatocytes expressing PNPLA3-I148M have more long-chain polyunsaturated fatty acids (PUFA) [17]. Mitsche et al. reported that hepatocytes expressing PNPLA3-I148M could transfer PUFA from triglyceride to phospholipid [18]. Phospholipid remodeling is associated with NAFLD susceptibility.

Although the research on the relationship between PNPLA3 gene polymorphism and NAFLD in children has attracted the attention of many researchers, the results vary from study to study. However, these studies were small sample sizes and had low statistical power which may lead to contradictory results. Hence, we performed this meta-analysis by combining the independent studies and estimating the overall effect to overcome the individual limitation and to draw more convinced conclusions.

In this study, by developing a retrieval strategy, literature quality evaluation according to the requirements of the Oxford Critical Appraisal Skill Program (Oxford CASP, 2004) [19], 9 literatures meeting the requirements were included for data extraction. We finally consolidated a total of 5 eligible studies to seek the relationship between PNPLA3 738,409 locus gene polymorphism and NAFLD susceptibility in children. The random effect model was used to conduct combined analysis of the data. Allele model, dominant gene model, recessive gene model and superdominant gene model of PNPLA3 738,409 locus were analyzed in this study, and the results showed that PNPLA3 738,409 locus gene polymorphism was significantly associated with NAFLD susceptibility in children. 4 literatures studied the relationship between PNPLA3 738,409 locus gene polymorphism and NAFLD severity in children. The fixed effect model was used to conduct combined analysis of the data. Due to data limitations, the recessive gene model of PNPLA3 738,409 locus was analyzed, and the results showed that PNPLA3 738,409 locus gene polymorphism was significantly associated with NASH in children.

At the same time, there are still some limitations in this article. First, due to the lack of a unified document quality evaluation standard, the included articles are subjectively selected and evaluated, which may affect the stability of the meta-analysis results. Second, since meta-analysis itself is a retrospective study, there is a degree of bias. Due to these limitations, we still need to expand the sample size to further and systematically evaluate case-control studies. In addition, this meta-analysis only involved single-factor studies, and did not include the interaction of PNPLA3 gene polymorphism with obesity [20] and breastfeeding [21] for hours, and the interaction of the above factors may affect the susceptibility of NAFLD in children.

## Conclusion

In summary, the polymorphism of PNPLA3 738,409 locus gene polymorphism is not only related to the susceptibility of NAFLD in children, but also related to its severity. Because NAFLD in children has no specific clinical manifestations, and ultrasound is not sensitive to the diagnosis of NASH, further studies can be conducted to evaluate whether PNPLA3 738,409 G/C gene polymorphism can be screened for early diagnosis of childhood NAFLD and early evaluation of NAFLD severity.

## Supplementary information

**Additional file 1: Supplementary Fig. 1.** Sensitivity analysis of the relationship between PNPLA3 rs738409 G/C and NADLD in children in the dominant model(GG + GC vs CC). **Supplementary Fig. 2.** Sensitivity analysis of the relationship between PNPLA3 rs738409 G/C and NAFLD in children in the allele model (G vs C). **Supplementary Fig. 3.** Sensitivity analysis of the relationship between PNPLA3 rs738409 G/C and NAFLD in children in the recessive gene model (GG vs CG + CC). **Supplementary Fig. 4.** Sensitivity analysis of the relationship between PNPLA3 rs738409 G/C and NAFLD in children in the superdominant model (GG + CC vs CC). **Supplementary Fig. 5.** Sensitivity analysis of the relationship between PNPLA3 rs738409 G/C and NASH in children in the recessive gene model (GG vs CG + CC). **Supplementary Fig. 6.** Egger’s funnel plot of the relationship between PNPLA3 rs738409 G/C and NADLD in children in the dominant model(GG + GC vs CC). **Supplementary Fig. 7.** Funnel plot of the relationship between PNPLA3 rs738409 G/C and NADLD in children in the dominant model(GG + GC vs CC). **Supplementary Fig. 8.** Egger’s funnel plot of the relationship between PNPLA3 rs738409 G/C and NAFLD in children in the allele model (G vs C). **Supplementary Fig. 9.** Funnel plot of the relationship between PNPLA3 rs738409 G/C and NAFLD in children in the allele model (G vs C). **Supplementary Fig. 10.** Egger’s funnel plot of the relationship between PNPLA3 rs738409 G/C and NAFLD in children in the recessive gene model (GG vs CG + CC). **Supplementary Fig. 11.** Funnel plot of the relationship between PNPLA3 rs738409 G/C and NAFLD in children in the recessive gene model (GG vs CG + CC). **Supplementary Fig. 12.** Egger’s funnel plot of the relationship between PNPLA3 rs738409 G/C and NAFLD in children in the superdominant model (GG + CC vs CC). **Supplementary Fig. 13.** Funnel plot of the relationship between PNPLA3 rs738409 G/C and NAFLD in children in the superdominant model (GG + CC vs CC). **Supplementary Fig. 14.** Egger’s funnel plot of the relationship between PNPLA3 rs738409 G/C and NASH in children in the recessive gene model (GG vs CG + CC). **Supplementary Fig. 15.** Funnel plot of the relationship between PNPLA3 rs738409 G/C and NASH in children in the recessive gene model (GG vs CG + CC)

## Data Availability

All data generated or analyzed during this study are included in this published article.

## Abbreviations

NAFLD: Nonalcoholic fatty liver disease
PNPLA3: Patatin-like phospholipase domain containing 3
NAFL: Nonalcoholic fatty liver
NASH: Nonalcoholic steatohepatitis
BMI: Body mass index
HWE: Hardy-Weinberg equilibrium
