# Supplementary material for: Association of PNPLA3 rs738409 G/C gene polymorphism with nonalcoholic fatty liver disease in children: a meta-analysis
Source: BMC Med Genet. 2020 Aug 18;21:163. doi: 10.1186/s12881-020-01098-8 (PMC7433068; doi:10.1186/s12881-020-01098-8)
Supplement: Supplementary file 1 — Additional file 1: Supplementary Fig. 1. Sensitivity analysis of the relationship between PNPLA3 rs738409 G/C and NADLD in children in the dominant model(GG + GC vs CC). Supplementary Fig. 2. Sensitivity analysis of the relationship between PNPLA3 rs738409 G/C and NAFLD in children in the allele model (G vs C). Supplementary Fig. 3. Sensitivity analysis of the relationship between PNPLA3 rs738409 G/C and NAFLD in children in the recessive gene model (GG vs CG + CC). Supplementary Fig. 4. Sensitivity analysis of the relationship between PNPLA3 rs738409 G/C and NAFLD in children in the superdominant model (GG + CC vs CC). Supplementary Fig. 5. Sensitivity analysis of the relationship between PNPLA3 rs738409 G/C and NASH in children in the recessive gene model (GG vs CG + CC). Supplementary Fig. 6. Egger’s funnel plot of the relationship between PNPLA3 rs738409 G/C and NADLD in children in the dominant model(GG + GC vs CC). Supplementary Fig. 7. Funnel plot of the relationship between PNPLA3 rs738409 G/C and NADLD in children in the dominant model(GG + GC vs CC). Supplementary Fig. 8. Egger’s funnel plot of the relationship between PNPLA3 rs738409 G/C and NAFLD in children in the allele model (G vs C). Supplementary Fig. 9. Funnel plot of the relationship between PNPLA3 rs738409 G/C and NAFLD in children in the allele model (G vs C). Supplementary Fig. 10. Egger’s funnel plot of the relationship between PNPLA3 rs738409 G/C and NAFLD in children in the recessive gene model (GG vs CG + CC). Supplementary Fig. 11. Funnel plot of the relationship between PNPLA3 rs738409 G/C and NAFLD in children in the recessive gene model (GG vs CG + CC). Supplementary Fig. 12. Egger’s funnel plot of the relationship between PNPLA3 rs738409 G/C and NAFLD in children in the superdominant model (GG + CC vs CC). Supplementary Fig. 13. Funnel plot of the relationship between PNPLA3 rs738409 G/C and NAFLD in children in the superdominant model (GG + CC vs CC). Supplementary Fig. 14. Egger’s fu [file 12881_2020_1098_MOESM1_ESM.pdf]

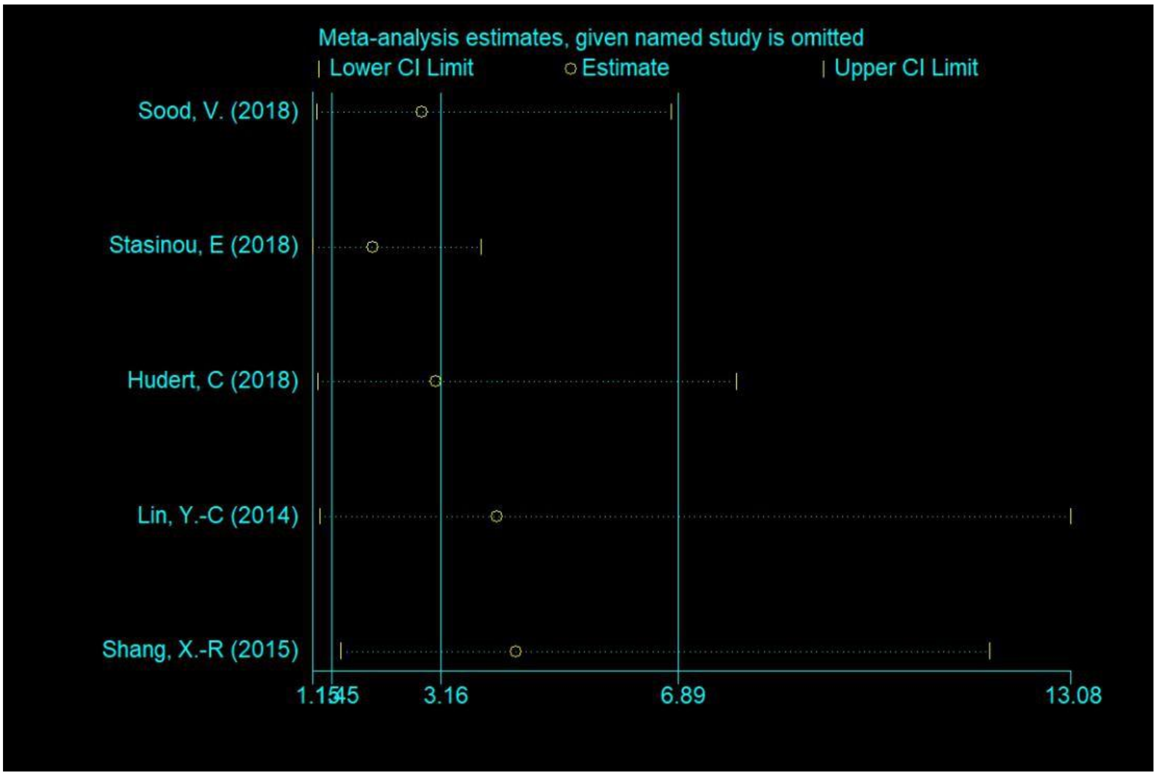


Supplementary Figure 1 Sensitivity analysis of the relationship between PNPLA3 rs738409 G/C and NADLD in children in the dominant model(GG+GC vs CC)

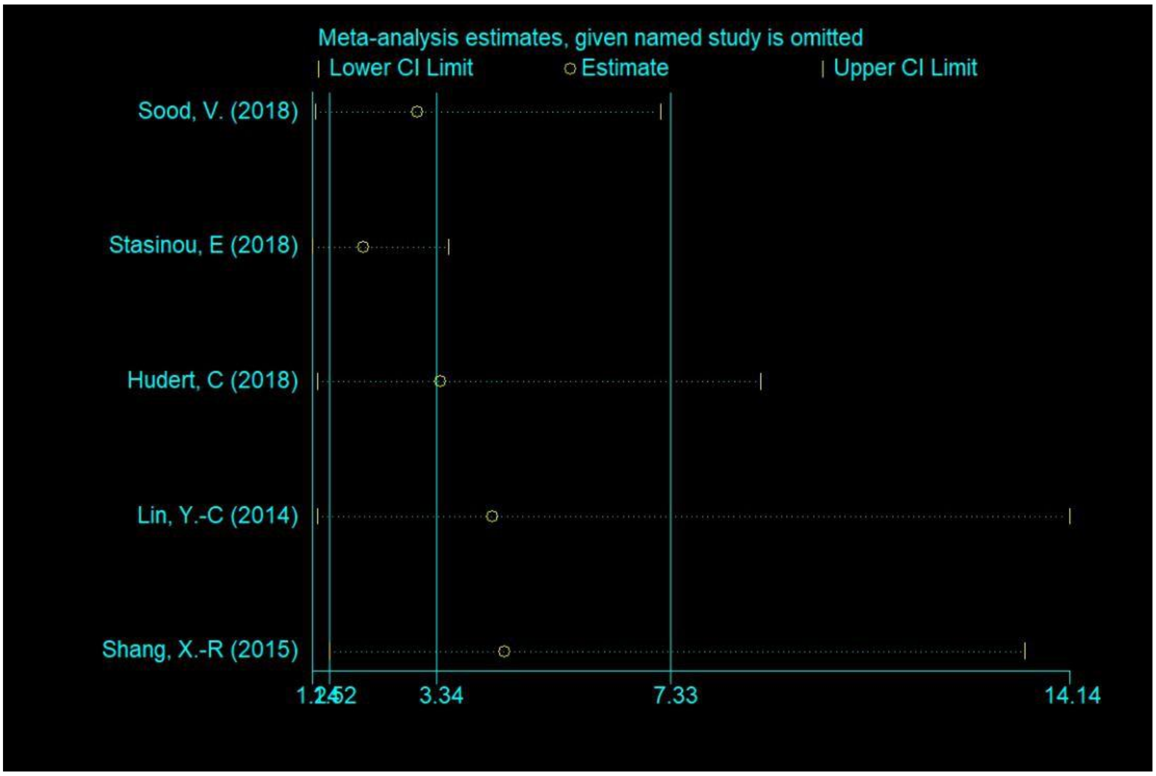


Supplementary Figure 2 Sensitivity analysis of the relationship between PNPLA3 rs738409 G/C and NAFLD in children in the allele model (G vs C)

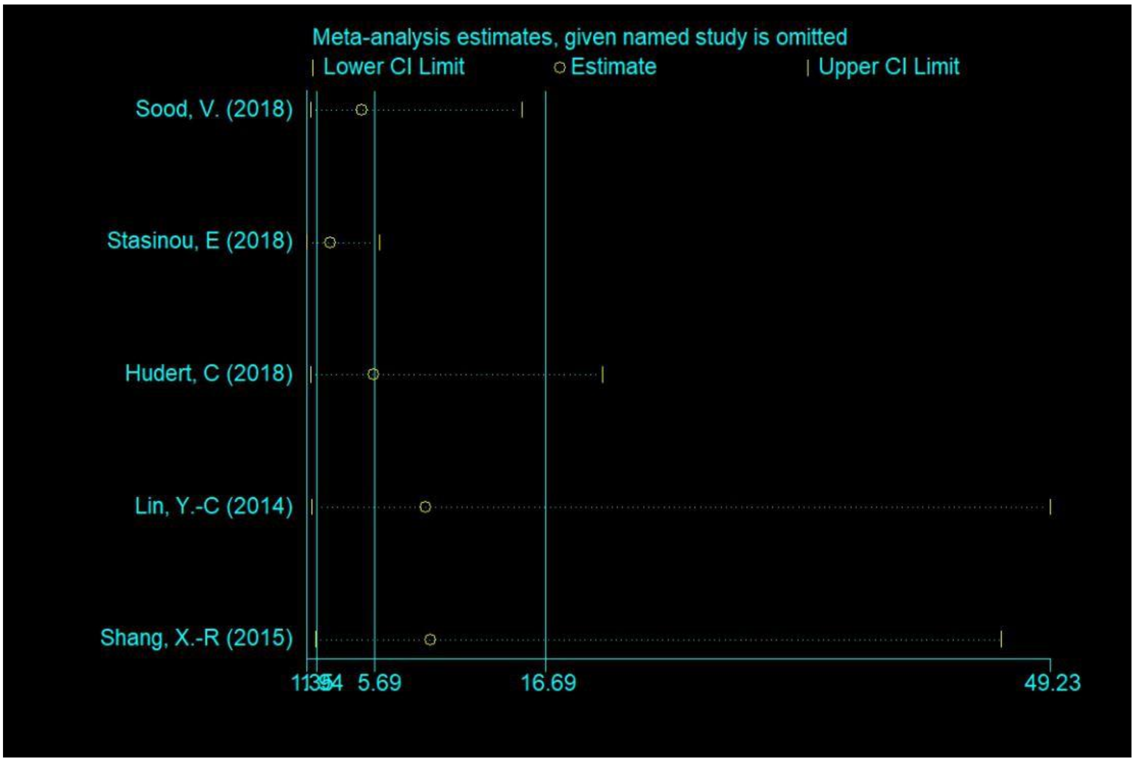


Supplementary Figure 3 Sensitivity analysis of the relationship between PNPLA3 rs738409 G/C and NAFLD in children in the recessive gene model (GG vs CG+CC)

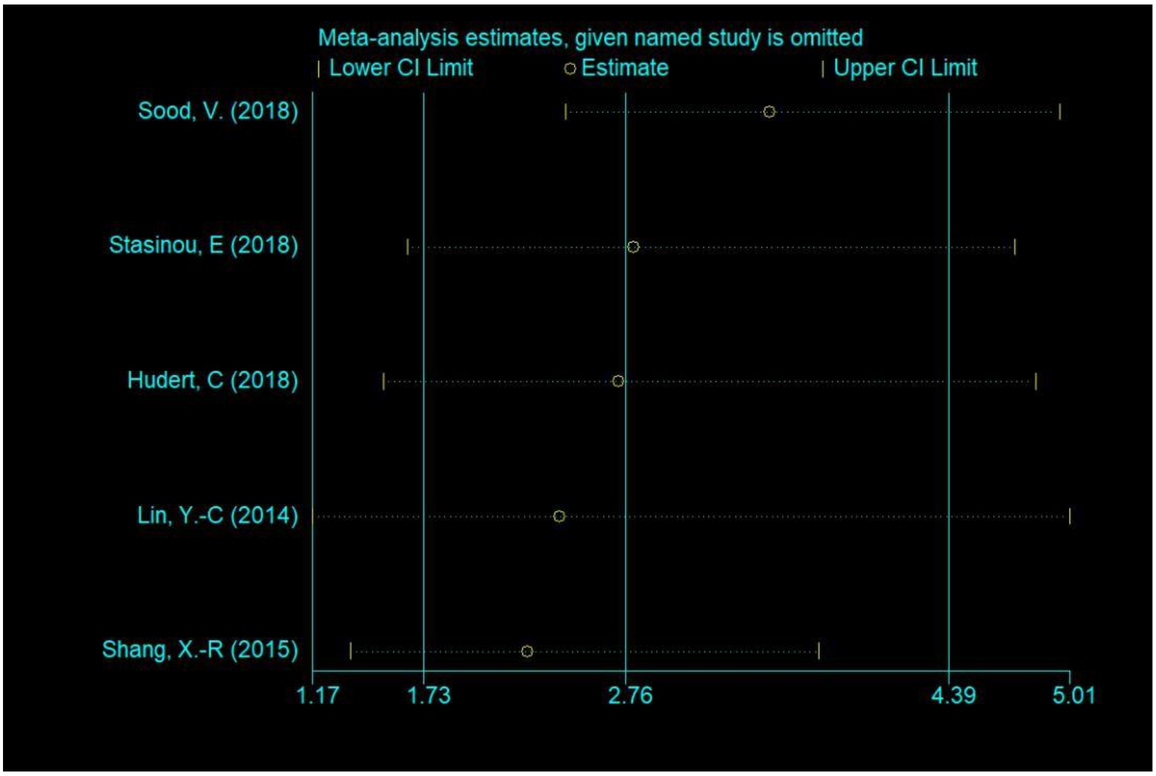


Supplementary Figure 4 Sensitivity analysis of the relationship between PNPLA3 rs738409 G/C and NAFLD in children in the superdominant model (GG+CC vs CC)

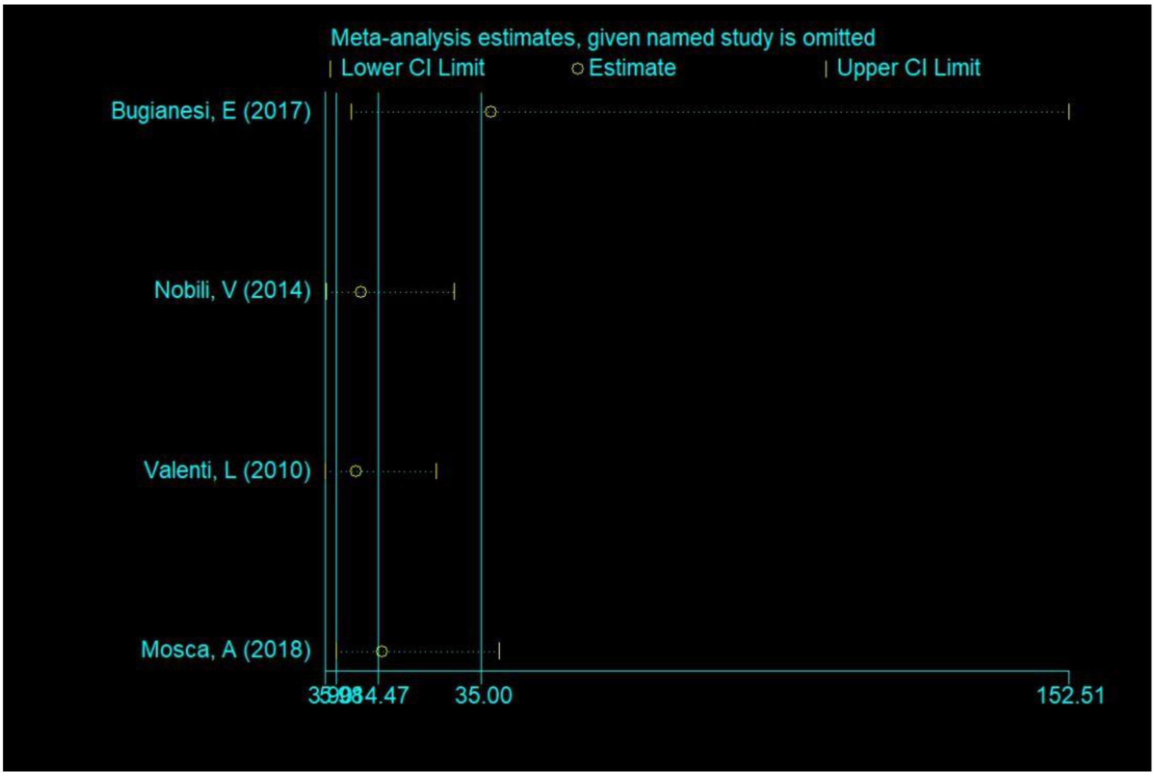


Supplementary Figure 5 Sensitivity analysis of the relationship between PNPLA3 rs738409 G/C and NASH in children in the recessive gene model (GG vs CG+CC)

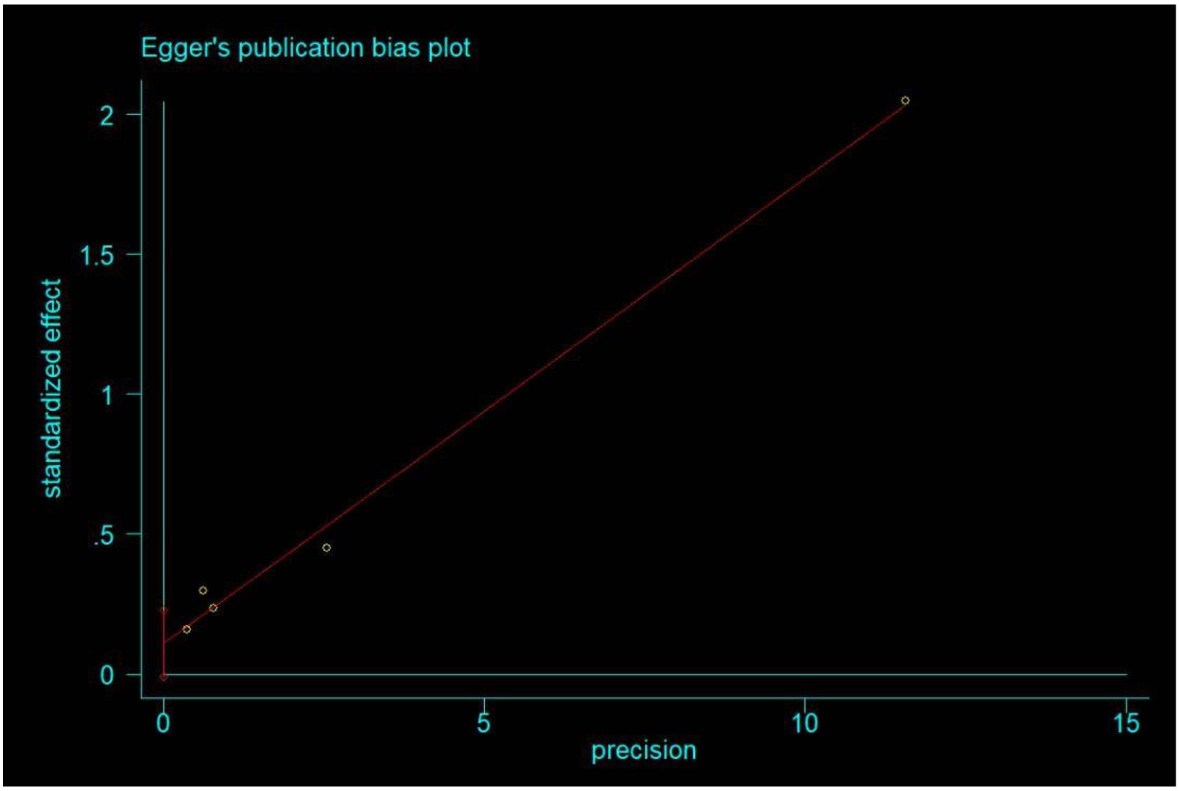


Supplementary Figure 6 Egger's funnel plot of the relationship between PNPLA3 rs738409 G/C and NADLD in children in the dominant model(GG+GC vs CC)

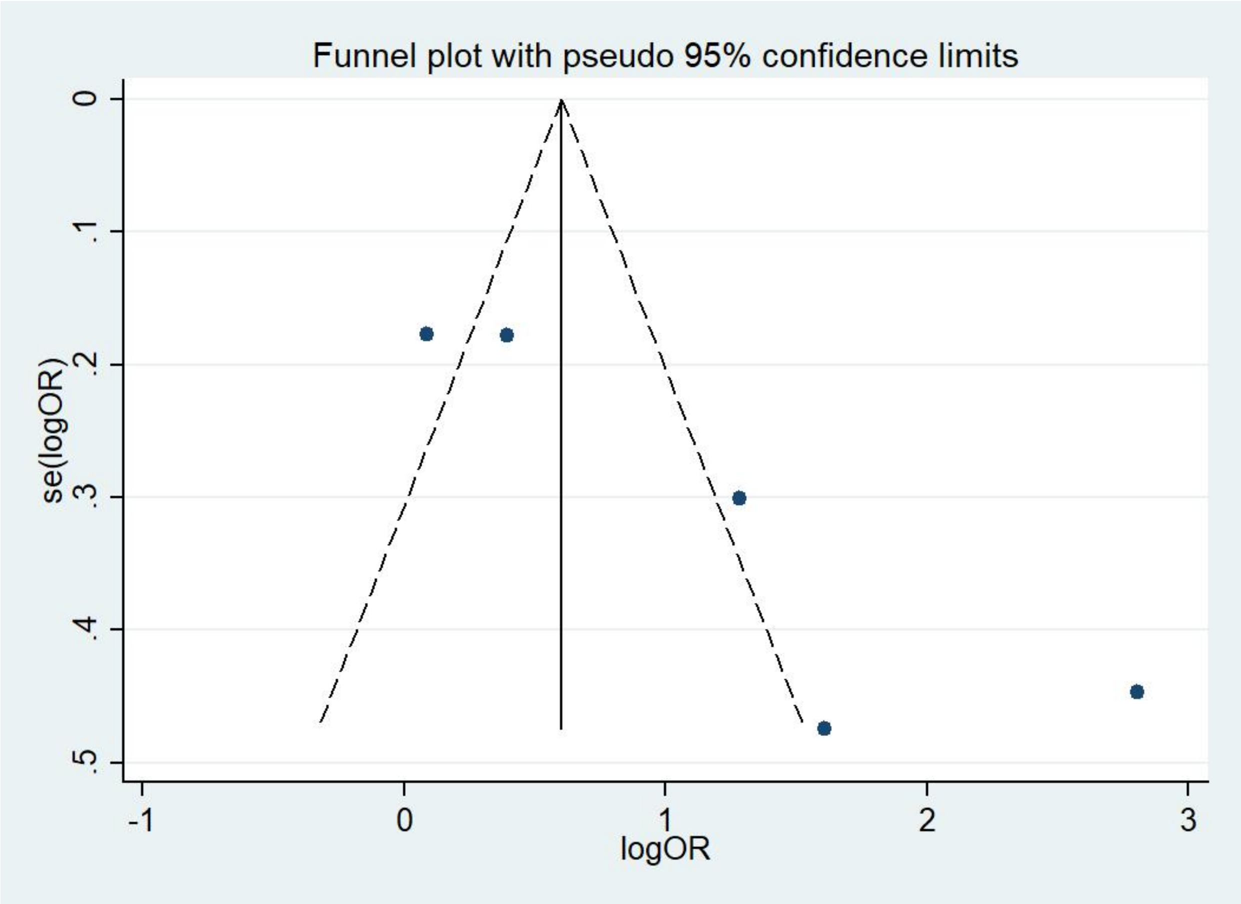


Supplementary Figure 7 Funnel plot of the relationship between PNPLA3 rs738409 G/C and NADLD in children in the dominant model(GG+GC vs CC)

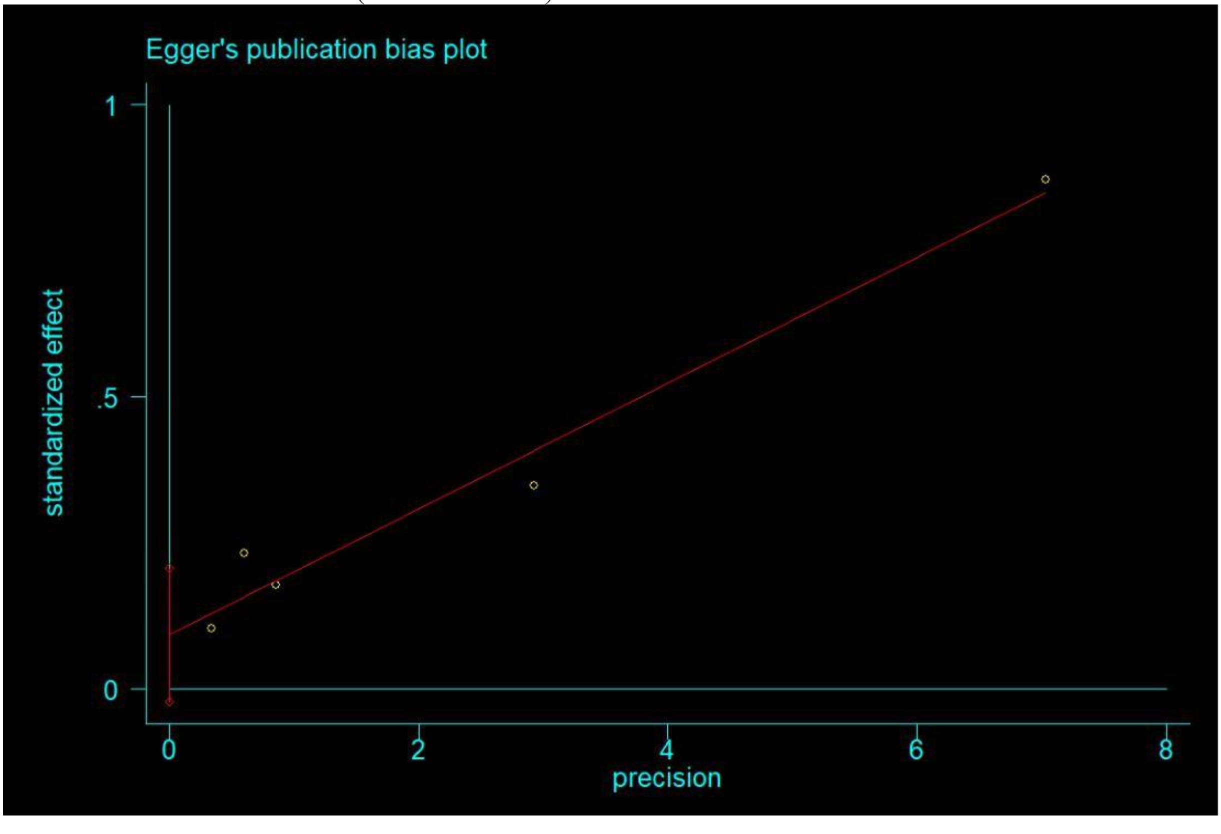


Supplementary Figure8 Egger's funnel plot of the relationship between PNPLA3 rs738409 G/C and NAFLD in children in the allele model (G vs C)

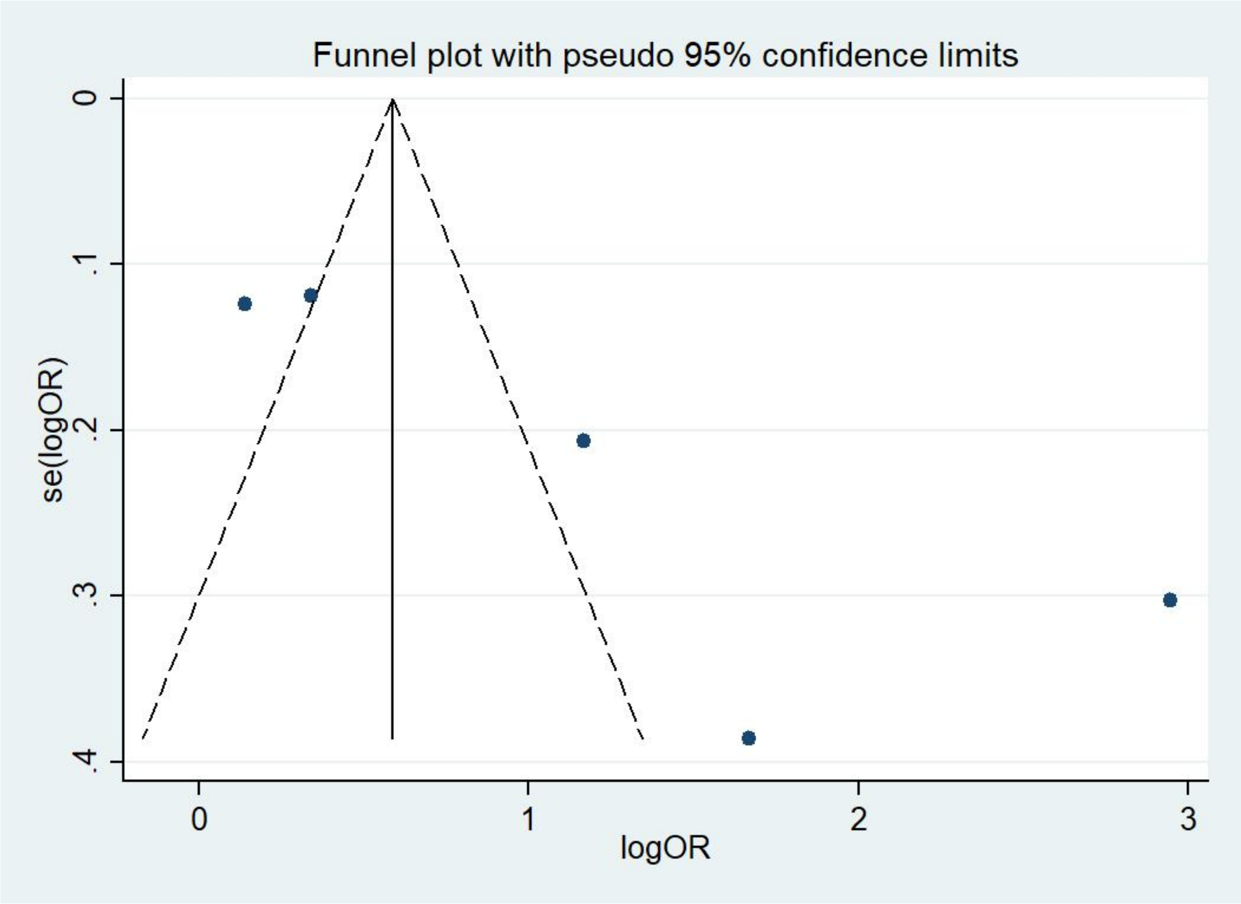


Supplementary Figure9 Funnel plot of the relationship between PNPLA3 rs738409 G/C and NAFLD in children in the allele model (G vs C)

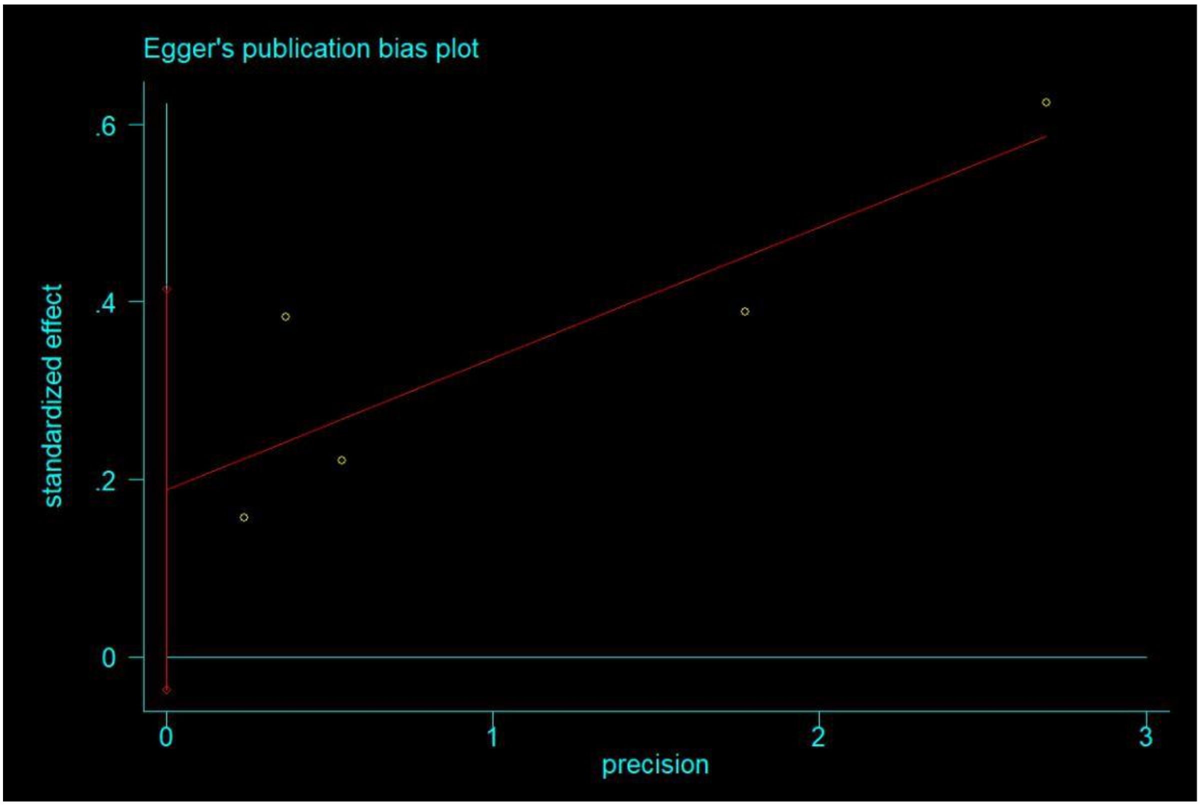


Supplementary Figure 10 Egger's funnel plot of the relationship between PNPLA3 rs738409 G/C and NAFLD in children in the recessive gene model (GG vs CG+CC)

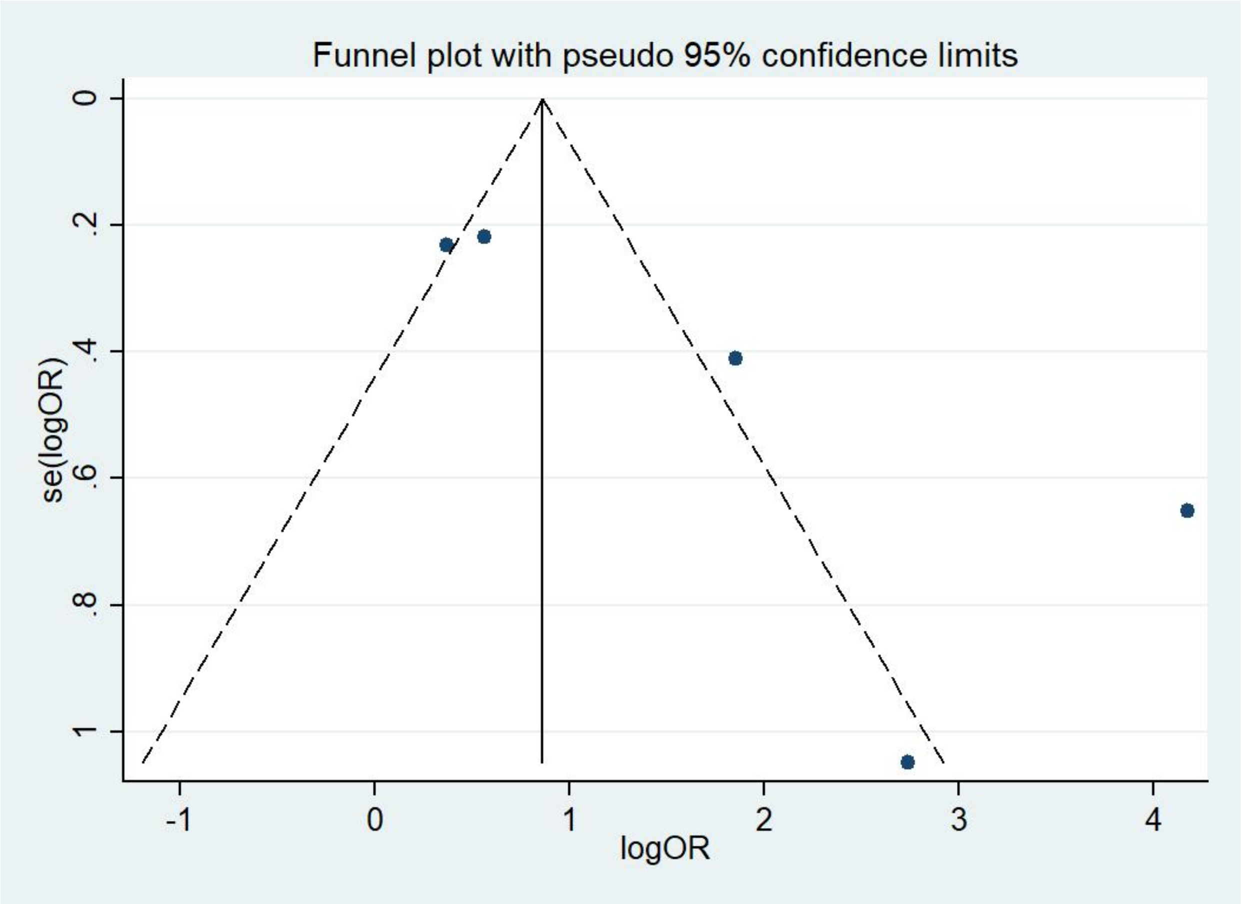


Supplementary Figure 11 Funnel plot of the relationship between PNPLA3 rs738409 G/C and NAFLD in children in the recessive gene model (GG vs CG+CC)

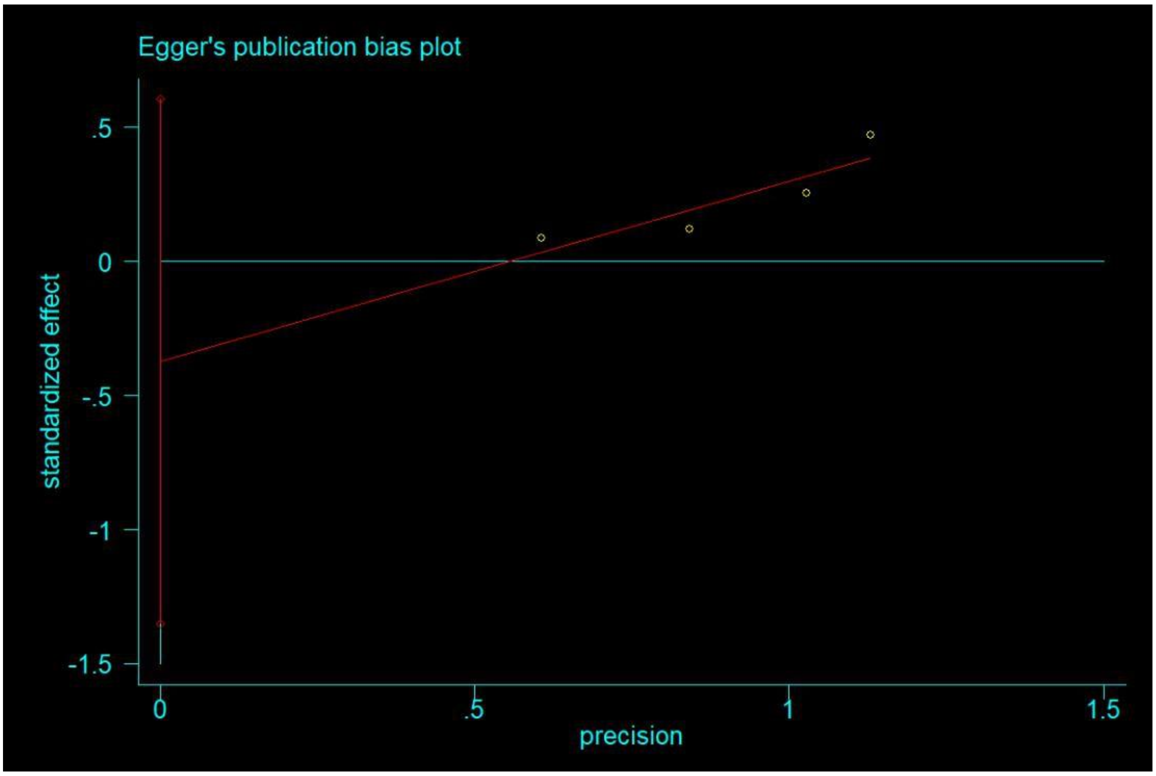


Supplementary Figure 12 Egger's funnel plot of the relationship between PNPLA3 rs738409 G/C and NAFLD in children in the superdominant model (GG+CC vs CC)

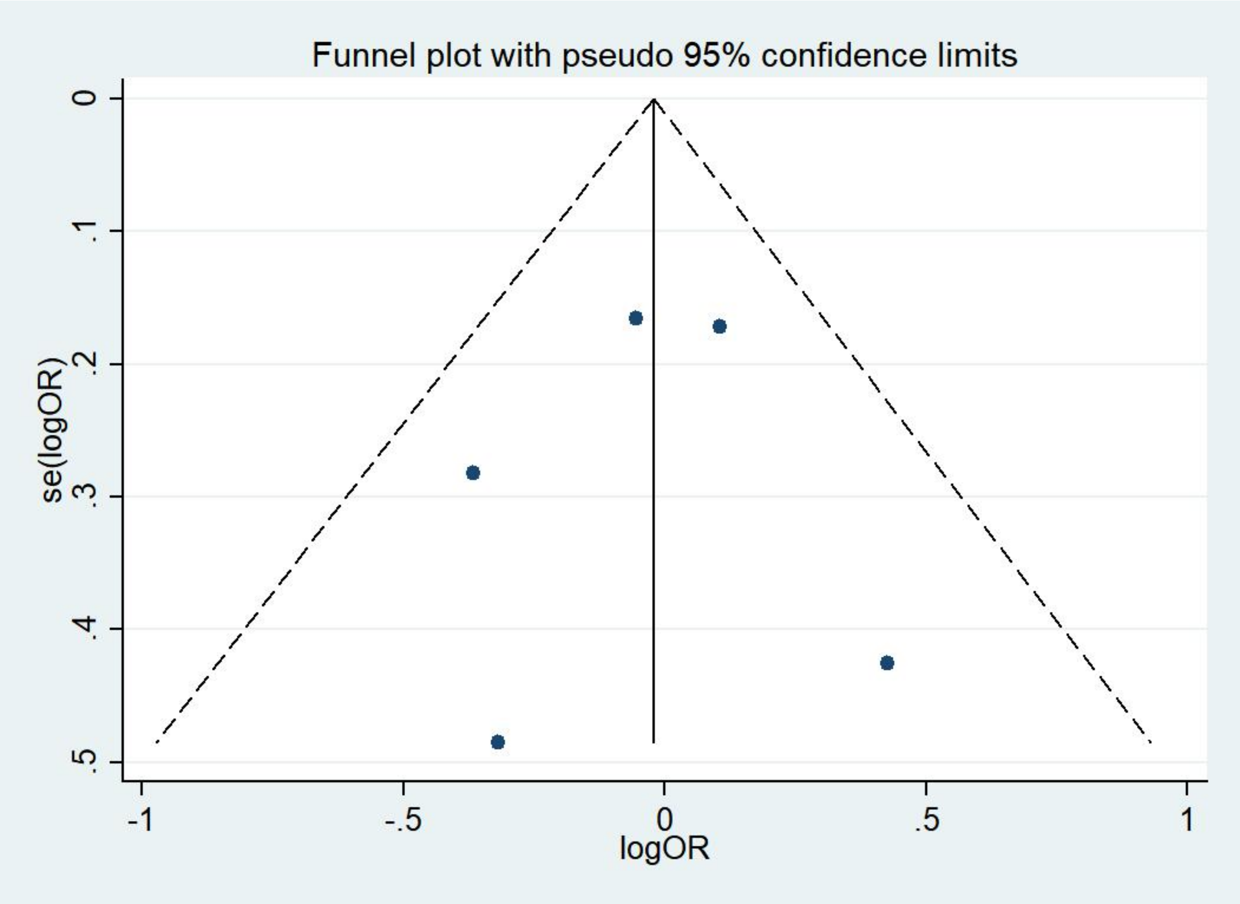


Supplementary Figure 13 Funnel plot of the relationship between PNPLA3 rs738409 G/C and NAFLD in children in the superdominant model (GG+CC vs CC)

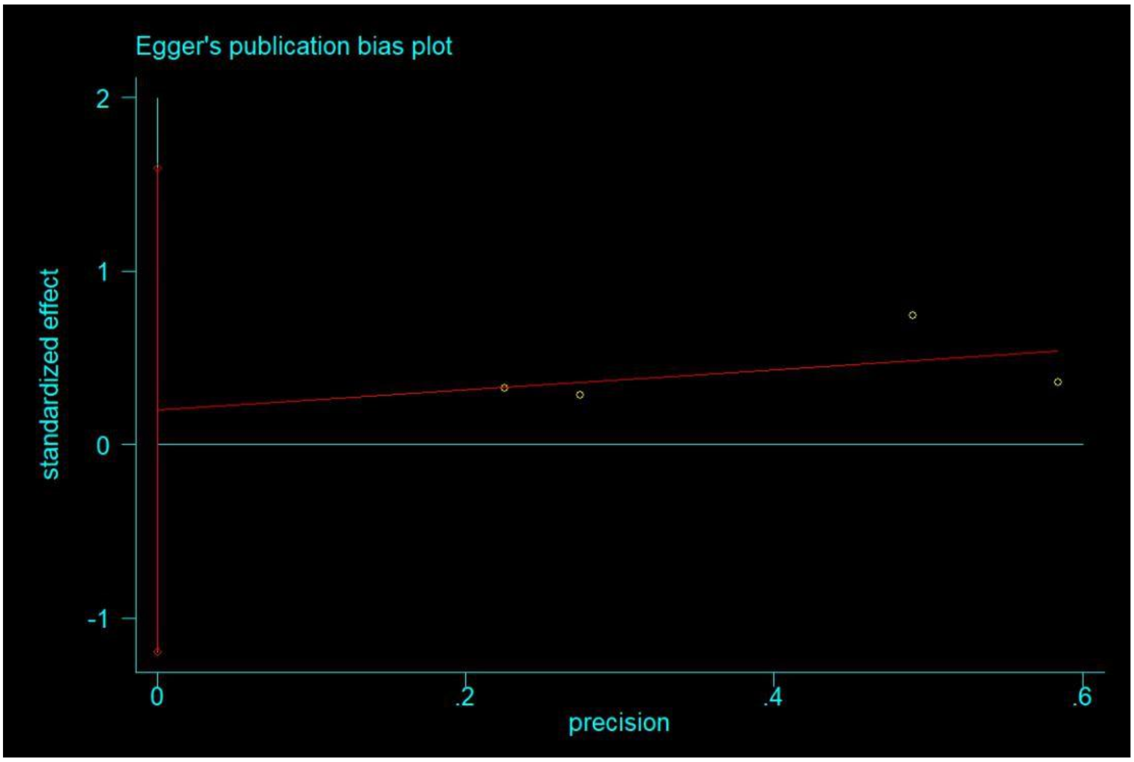


Supplementary Figure 14 Egger's funnel plot of the relationship between PNPLA3 rs738409 G/C and NASH in children in the recessive gene model (GG vs CG+CC)

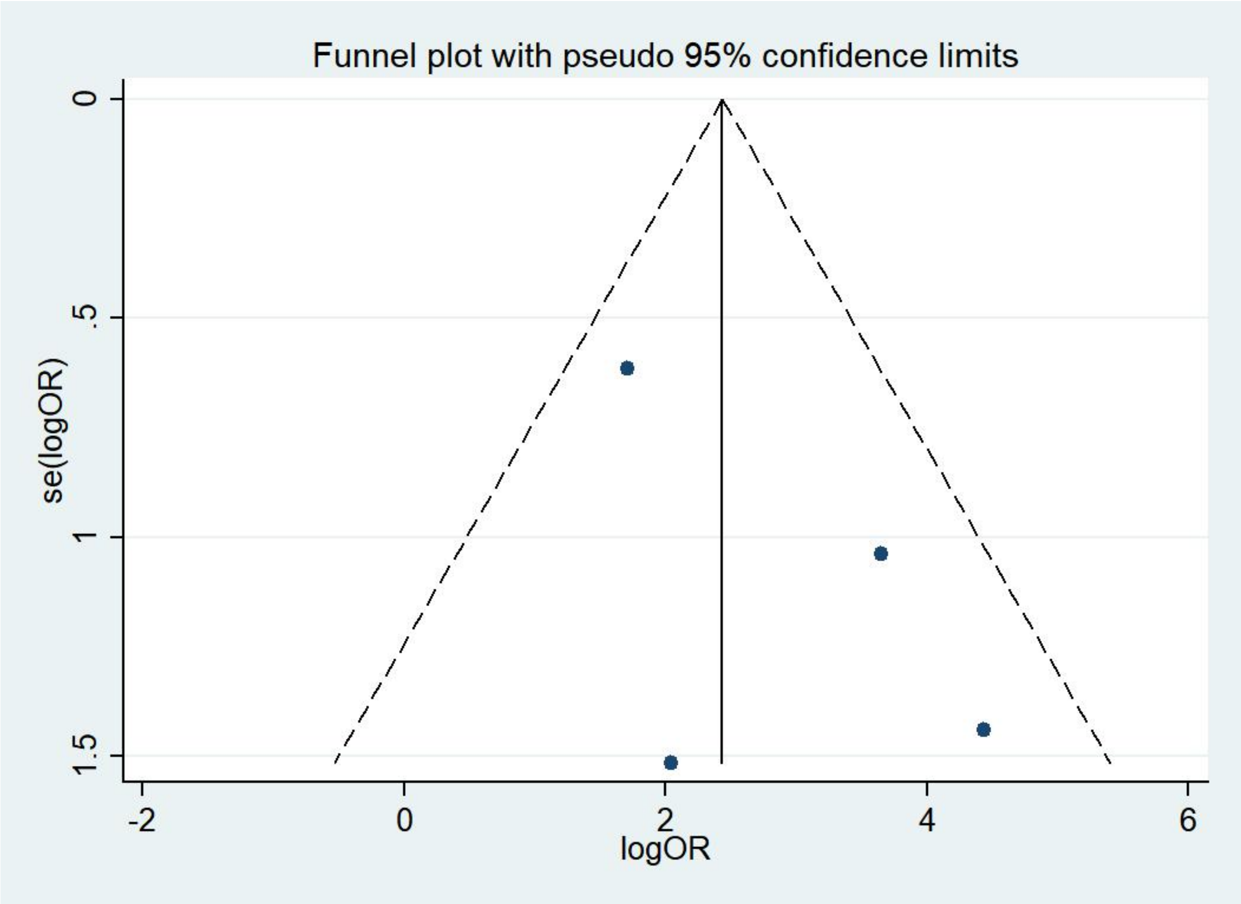


Supplementary Figure 15 Funnel plot of the relationship between PNPLA3 rs738409 G/C and NASH in children in the recessive gene model (GG vs CG+CC)
